# Local Th17/IgA immunity correlate with protection against intranasal infection with *Streptococcus pyogenes*

**DOI:** 10.1371/journal.pone.0175707

**Published:** 2017-04-17

**Authors:** Rasmus Mortensen, Dennis Christensen, Lasse Bøllehuus Hansen, Jan Pravsgaard Christensen, Peter Andersen, Jes Dietrich

**Affiliations:** 1 Statens Serum Institut, Department of Infectious Disease Immunology, Copenhagen, Denmark; 2 University of Copenhagen, Department of Immunology and Microbiology, Copenhagen, Denmark; 3 Rigshospitalet, Department of Growth and Reproduction, University of Copenhagen, Copenhagen, Denmark; University of South Dakota, UNITED STATES

## Abstract

*Streptococcus pyogenes* (group A streptococcus, GAS) is responsible for a wide array of infections. Respiratory transmission via droplets is the most common mode of transmission but it may also infect the host via other routes such as lesions in the skin. To advance the development of a future vaccine against GAS, it is therefore important to investigate how protective immunity is related to the route of vaccine administration. To explore this, we examined whether a parenterally administered anti-GAS vaccine could protect against an intranasal GAS infection or if this would require locally primed immunity. We foundd that a parenteral CAF01 adjuvanted GAS vaccine offered no protection against intranasal infection despite inducing strong systemic Th1/Th17/IgG immunity that efficiently protected against an intraperitoneal GAS infection. However, the same vaccine administered via the intranasal route was able to induce protection against repeated intranasal GAS infections in a murine challenge model. The lack of intranasal protection induced by the parenteral vaccine correlated with a reduced mucosal recall response at the site of infection. Taken together, our results demonstrate that locally primed immunity is important for the defense against intranasal infection with *Streptococcus pyogenes*.

## Introduction

Group A streptococcus (GAS; *Streptococcus pyogenes*) is a human pathogen causing hundreds of millions of infections each year throughout the world. Acute benign *Streptococcus pyogenes* infections may present as both pharyngitis and superficial skin infections. GAS can also be invasive and result in severe conditions such as necrotizing fasciitis, myositis and streptococcal toxic shock syndrome. Finally, patients may also develop immune-mediated post-streptococcal sequelae, acute rheumatic fever and glomerulonephritis. No vaccines exist against this pathogen. Moreover, two largely unanswered key questions concerning GAS immunity are: 1) What constitutes the correlates of protection, in particular regarding cellular immunity, and 2) to which degree is vaccine-induced protection against an infection with GAS dependent on the route of vaccine administration (i.e. systemic vs. mucosal administration). In the present study, we asked whether a Th1/Th17 promoting adjuvant (CAF01 [1]) formulated with GAS antigen (and administered by the parenteral route) could induce protection against an intranasal infection with GAS. We chose the adjuvant CAF01 for its immunological profile, which involve both induction of IgG as well as Th1/Th17 T cell immunity [2, 3]. Concerning the importance of IgG, there is ample evidence that antibodies play a role in protective immunity [4–9]. These can contribute to protection by activating complement deposition via the classical pathway, by direct opsonization mediated via Fc receptors on phagocytes and by neutralization of virulence factors and adhesins. In humans, GAS exposure leads to induction of antibodies against both the M protein and non-M proteins [10–14], and we recently showed that the IgG1 and IgG3 subclasses dominate this response [15]. Regarding the role of T cells, there have been indications that they do participate in protective anti-GAS immunity. It is known that whole GAS bacteria activate a Th1/Th17 promoting response in human macrophages and dendritic cells [16–19] and that mice develop Th1/Th17 immunity when inoculated intranasally with live GAS bacteria [20, 21]. In particular, animal studies have indicated that Th17 cells possess a protective capacity against a GAS infection [21–23].

Regarding the issue of vaccine administration route, early studies in humans have indicated importance for local immunity against mucosal infection [24]. To explore this further, we used a mouse GAS infection model to test the protective ability of a parenteral vaccine based on the adjuvant CAF01. We did not use the common infection model where the animals receive a lethal intranasal dose, as we believe that such a model might be suboptimal in terms of examining the role of T cells that require more time to exert their effector function compared to antibodies. Instead, we developed a repeated infection model where the animals received several sub lethal infections. In this model, we tested the ability of a parenteral and intranasal anti-GAS Th1/Th17 inducing vaccine to protect against an intranasal infection with GAS and correlated any protection with the early recall response induced at the site of infection.

## Materials and methods

### Animals

Female CB6F1 hybrid mice (offspring of female BALB/c and male C57BL/6 mice) at 6–8 weeks of age were purchased at Envigo Laboratories (The Netherlands) and randomly assigned to cages at the animal facility at Statens Serum Institut upon arrival. Animals were rested for one week before any experimental manipulation and they were allowed free access to water and food throughout the experiment. Experiments were conducted in accordance with the regulations set forward by the Danish Ministry of Justice and animal protection committees by Danish Animal Experiments Inspectorate Permit 2004-561-868 (of January 7, 2004) and in compliance with European Community Directive 2010/63/EU of the European parliament and of the council of 22 September 2010 on the protection of animals used for scientific purposes, as well as Directive 86/609 and the U.S. Association for Laboratory Animal Care recommendations for the care and use of laboratory animals. The experiments were approved by a local animal protection committee at the Statens Serum Institut, IACUC, headed by DVM Kristin E. Engelhart Illigen.

### Streptococcal infection models

Mice received a systemic (homologous) challenge by injecting a lethal dose of MGAS5005 (serotype M1; 1–1.5 x 10^7^ CFU/mouse) into the peritoneum (i.p. injection). Mice were monitored individually once every 2–4 hours over a period of 24 hours and euthanized when they reached defined humane endpoints. A validated clinical scoring system derived from the consolidation Act on Experimental Animals (BEK-88 30.01.2013) was used in the health monitoring and the staff were experienced with the use of this system running from 0–4. 0: The mouse was unaffected. 1: The mouse was slightly affected (e.g. incipient hourglass figure (abdominal pain) or that the fur was starting to bristle) 2: The mouse was moderately affected (e.g. hourglass figure, bristling fur or a little less mobile) 3: The mouse was clearly affected (bristling fur, eyes partially closed, buckled back, hourglass figure, reduced mobility and changed breathing) 4: The mouse was severely affected (e.g. the fur bristled a lot, the mouse was cold, immobile and doubled up and the eyes were closed). Mice were euthanized at the score of 4. In the repeated infection model, mice were infected four times with a medium dose of MGAS5005 bacteria (10^6^ CFU) via the i.n. route, at 2–3 weeks interval. In other experiments mice were infected i.n. with one dose of MGAS5005 bacteria (5 x 10^7^ CFU) via the i.n. route. The mice were anesthetized shortly in an inhalation chamber with isoflurane and infected by administering 15 μl of the bacterial suspension in each nostril. Following infection, the bacterial numbers in throat swaps were determined at day 1–7 post infection. For some infections we also determined bacterial numbers in nasal fluid, NALT and lung.

### Antigens, adjuvants and immunizations

We used heat killed GAS bacteria (HGAS) as model antigen throughout this study. GAS colonies were grown on blood agar plates and harvested in Tris-HCL buffer pH 7.5 before determining the bacterial concentration by plating. Bacteria were diluted to 10^9^ CFU/ml and killed by heating the suspension for 120 min at 60°C. HGAS/Tris-HCl pH 7.5 was stored in aliquots at -20°C until used. For immunizations, liposomes of dimethyldioctadecylammonium (DDA) and trehalose 6,6′-dibehenate (TDB) (DDA/TDB; CAF01) [1] was used as adjuvant at a dose of 250/50 μg per immunization. Adjuvants were mixed with 10^8^ HGAS by vortexing and mice were immunized by the subcutaneous route (s.c., injection volume 200 μl) at the base of the tail or the nasal route (i.n., inhalation volume 2 x 20 μl). Two to three vaccinations were given with 2–3 weeks spacing and serum was collected two weeks after the last immunization and stored at -20°C prior to determination of antigen specific antibodies.

### Detection of specific antibodies by ELISA

Maxisorp micro titer plates (Nunc, Maxisorp) were coated with 2 x 10^7^ HGAS/ml in carbonate buffer pH 9.6 (SSI Diagnostica) over night at 4°C. The day after, free bindingsites were blocked with 3% skimmed milk (w/v) + 0.05% (v/v) Tween-20 in PBS for 1.5 hour at room temperature. After three washes, individual serum and lung samples were added in 10-fold serial dilutions in PBS containing 1% skimmed milk (w/v) + 0.05% (v/v) Tween-20 starting with a 10-fold dilution. Following 2 hours of incubation, plates were washed three times and incubated for 1 hour with HRP-conjugated secondary antibody. HRP-conjugated polyclonal anti-mouse IgG (Invitrogen) in a 1:20000 dilution and anti-mouse IgA (Zymed) in 1:5000 was used. Specific antibodies were detected by an enzyme reaction with TMB substrate after 5 washes. Reactions were stopped with 0.2 M H_2_SO_4_ after 30 min, and the optical density (OD) was measured at 450nm with 620nm correction.

### Lymphocyte cultures

Murine PBMCs were purified from blood in EDTA tubes using a density gradient. Mononuclear cells from the lung were isolated after a collagenase digestion. In brief, lungs were transferred to gentleMACS C Tubes (Miltenyi Biotec GmbH) containing 2 mL RPMI 1640 with 5% fetal calf serum (FCS) (Gibco; Invitrogen) and 0.8 mg/ml Collagenase type IV (Sigma) and dissociated with the gentleMACS™ Octo Dissociator (Miltenyi Biotec GmbHy). The collagenase treatment was performed at 37°C for 1h. Samples were returned to the gentleMACS Dissociator and dissociated gently followed by centrifugation at 700 x g for 5 minutes. Supernatants were used for antibody measurements and the pellet containing lung cells was homogenized through a 100μm cell-strainer (Falcon, Durham, NC) and washed twice in RPMI-1640 (Gibco Invitrogen). Splenocytes were prepared by pressing an excised spleen through the strainer. After washing, 2 x 10^5^ PBMCs, lung or spleen cells were incubated at 37°C/5% CO_2_ in round bottom 96-well microtiter plates (Nunc) in 200 μl RPMI-1640 supplemented with 5×10^−5^ M 2-mercaptoethanol, 1 mM glutamine, 1% pyruvate, 1% penicillin-streptomycin, 1% HEPES and 10% FCS. Stimulations were performed with 5 x 10^6^ HGAS/ml and culture supernatants in triplicates were harvested 3 days later for IFNγ and IL-17 ELISA as well as cytokine multiplex analysis. For intracellular cytokine staining (ICS) 1–2 x 10^6^ cells were cultured and stained as described below.

### Flow cytometry

Lung cells were incubated for 1 h in the presence of 1 μg/ml anti-CD28 (clone 37.51; BD Pharmingen) and anti-CD49d (clone 9C10 (MFR4.B); BD Pharmingen) followed by 5 h with 10 μg/ml brefeldin A (Sigma-Aldrich) and 1:1500 BD GolgiStop (BD Biosciences) at 37°C in an automated heater that cooled the cells to 4°C after incubation. The next day, cells were washed in FACS buffer (PBS containing 0.1% sodium azide and 1% FCS) and stained at 4°C for surface markers using anti-CD4-APC-eF780 (clone RM4-5; 1/600 dilution; eBioscience) and anti-CD44-FITC (clone IM7; 1/200 dilution; BD Bioscience). After 15–30 min of surface staining, cells were washed in FACS buffer, permeabilized using the Cytofix/Cytoperm kit (BD Pharmingen) according to the manufacturer’s instructions, and stained intracellularly with anti-IL-17-PerCP-Cy5.5 (clone eBio17B7; 1/200 dilution; eBioscience) and anti-IFNγ-PE-Cy7 (clone XMG1.2; 1/200, eBioscience) mAbs. Cells were subsequently washed, resuspended in FACS buffer and analyzed on a BD FACSCanto flow cytometer (BD Biosciences). In vivo staining with anti-CD45.2: Anti-CD45.2-FITC (clone 104; BD Pharmingen) was diluted to 10μg/ml in sterile Phosphate-buffered saline (PBS). 250μl of the aCD45.2 solution was injected i.v. into each mouse via the tail vein, three minutes before euthanization of the mice. Data was analyzed using FlowJo v10.0.7 for Windows.

### Cytokine ELISA and multiplex assays

The levels of secreted cytokines in culture supernatants were evaluated using standard sandwich ELISA for IL-17 and IFNγ as previously described [2] or a multiplex Meso Scale Discovery assay (MSD). The cytokine 6-plex electro-chemiluminescence MSD assay measuring IFNγ, TNFα, IL-2, IL-5, IL-10 and IL-17 was performed using a mouse MULTI-SPOT® 96-well 7-Spot TC Custom Plate Mouse 6-plex Kit from MSD according to the manufacturer’s instructions. Plates were read on the Sector Imager 2400 system, and calculation of cytokine concentrations in unknown samples was determined by 4-parameter logistic non-linear regression analysis of the standard curve.

### Bacterial strains and growth

GAS strain MGAS5005 (serotype M1) was grown at 37°C with 5% CO_2_ in Todd-Hewitt broth (SSI Diagnostica) or 5% blood agar (SSI Diagnostica) that was used as solid medium for determining the colony forming units (CFU)

### Statistical analysis

Immune responses (secreted IFNγ/IL-17, IgG/IgA EP, % IL-17 or IFNγ positive T cells) were compared by one-way ANOVA followed by Tukey's multiple comparison test of the means or by a t-test as indicated. Differences in Kaplan-Meier survival curves were evaluated by a Chi-square test. CFU levels were compared by one-way ANOVA followed by Tukey's multiple comparison test of the means or in the repeated infection model by a unpaired t test, Two-tailed. A value of p<0.05 was considered significant. All statistical analyses were carried out in GraphPad Prism version 6.05 (GraphPad Software Inc.)

## Results

### Parenteral immunization with a Th1/Th17/IgG inducing adjuvant effectively protects against systemic GAS

**Fig 1:**
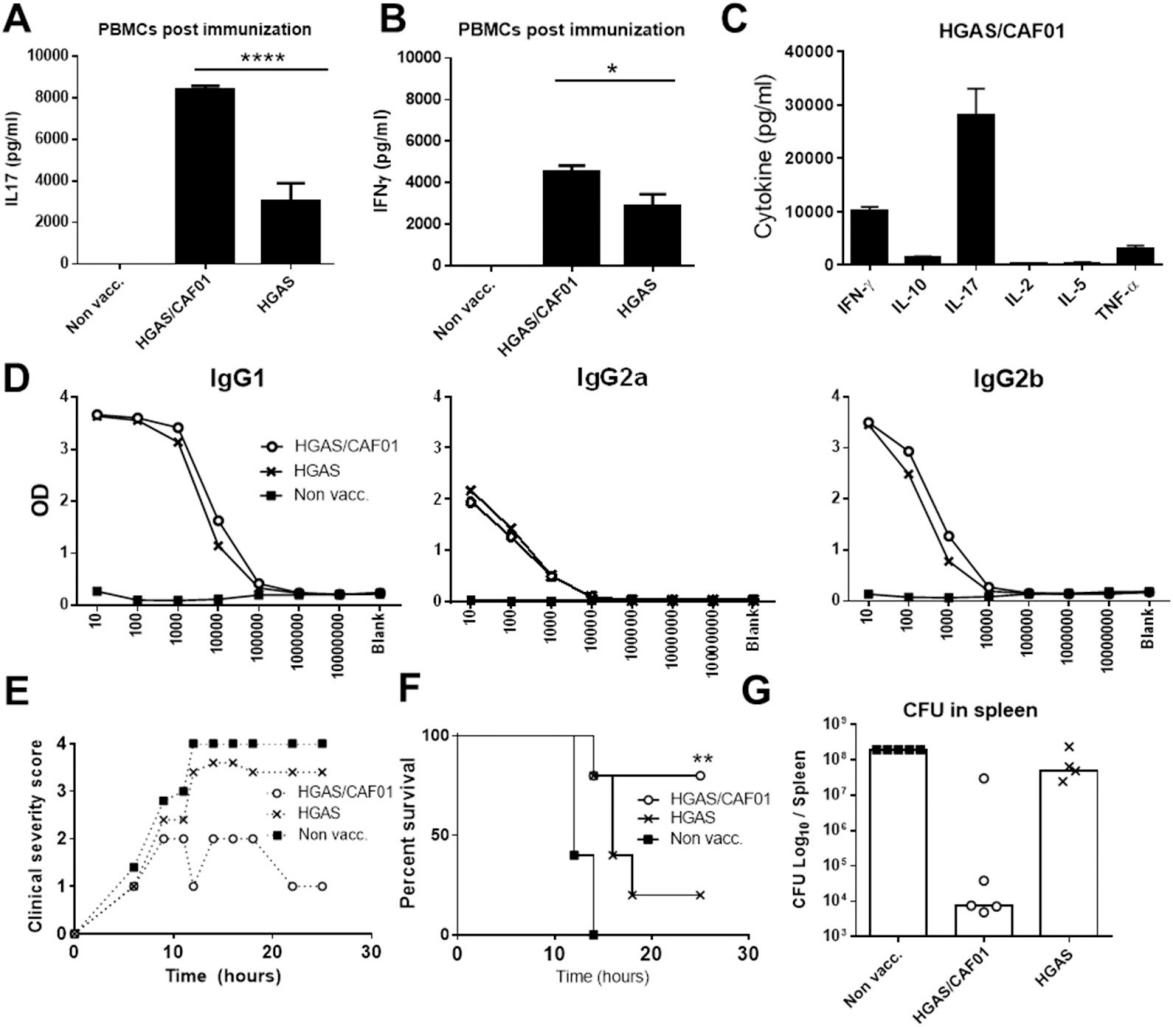
Heat inactivated GAS formulated in CAF01 induce protection against a systemic infection. Female CB6F1 mice (n = 8) were vaccinated 3 x s.c. with two weeks interval. Vaccines consisted of heat killed GAS (HGAS) either alone or formulated in CAF01 Two weeks after the last immunization, three animals were sacrificed and PBMCs were stimulated with HGAS for 72 h before measuring levels of **(A)** IFNγ and **(B)** IL-17 in culture supernatants by ELISA. An identical follow-up experiment (n = 10) was performed with the CAF01 adjuvant using multiplex cytokine analysis **(C)**. Bars indicate means ± SEM. Statistical significance was evaluated by an ANOVA followed by Tukey’s multiple comparison test using GraphPad Prism version 6.05. *p<0.05, **p<0.01, ***p<0.001 and ****p<0.0001. IgG responses in pooled serum samples were measured by antigen-specific ELISA **(D)**. Six weeks after the last immunization, the remaining mice (n = 5) were challenged with a lethal dose of MGAS5005 bacteria (1–1.5 10^7^ CFU/mouse) by i.p. injection. Mice were monitored over a period of 24 hours according to a validated clinical scoring system from 0–4 (**E**). When reaching a score of 4, mice were euthanized and data was plotted in Kaplan-Meier survival curves (**F**) (**p = 0.008 with a Chi-square test between the non-vaccinated animals and the CAF01 group). Bacterial numbers were measured in the spleen when mice were euthanized or at the end of the experiment 24 h post infection (**G**).

We first examined the protective capability of our model vaccine (administered as a parenteral vaccine) against a systemic infection with GAS. We used heat killed GAS (HGAS, strain MGAS5005, serotype M1; corresponding to 10^7^ CFU/mouse) as model antigen in combination with the Th1/Th17/IgG inducing adjuvant CAF01. Groups of 6–8 week old CB6F1 mice were vaccinated at week 0, 2, and 4 via the subcutaneous (s.c.) route. Two weeks following the final immunization, the immune response was examined in the blood. PBMCs were stimulated *in vitro* with HGAS for 72 hours, whereafter secretion of IL-17 and IFNγ was examined in culture supernatants. PBMCs from CAF01 vaccinated mice showed significantly increased secretion of both IL-17 and IFNγ compared to mice vaccinated with non-adjuvanted antigen (Fig 1A and 1B). A follow-up experiment demonstrated that IL-17 and IFNγwere the dominating cytokines secreted by antigen specific T cells of mice immunized with CAF01 (Fig 1C). Vaccinated mice developed anti-GAS serum IgG responses regardless of the use of adjuvant (Fig 1D).

Next, the mice were subjected to a systemic challenge by injecting a lethal dose of MGAS5005 (serotype M1; 1–1.5 x 10^7^ CFU/mouse) into the peritoneum (i.p. injection). Mice vaccinated with the Th1/Th17 adjuvant and HGAS showed increased survival and lower severity scoring (median of 1–2) (Fig 1E and 1F and S1 Fig) compared to both non-vaccinated mice and mice that received non-adjuvanted HGAS. A definition of the scores is given in materials and methods. Non-vaccinated, or HGAS vaccinated, mice also showed a higher clinical score after 10 hours (3–4), and the mice that were euthanized in these groups contained high bacterial numbers in the spleen, as expected (Fig 1G). All the non-vaccinated animals were euthanized within the first 14 hours. It was interesting that the lower CFU levels and increased survival, in the HGAS/CAF01 group correlated with an increased cellular response but not with an increased IgG response. This was also observed with another adjuvant, CpG, that also induced a selective increase in the Th1/Th17 response (S2 Fig).

Taken together, our data demonstrated that a s.c. administered vaccine based on HGAS and the adjuvant CAF01 effectively protected mice from an i.p infection with GAS.

### Systemic immunity does not protect against intranasal infection with GAS

**Fig 2:**
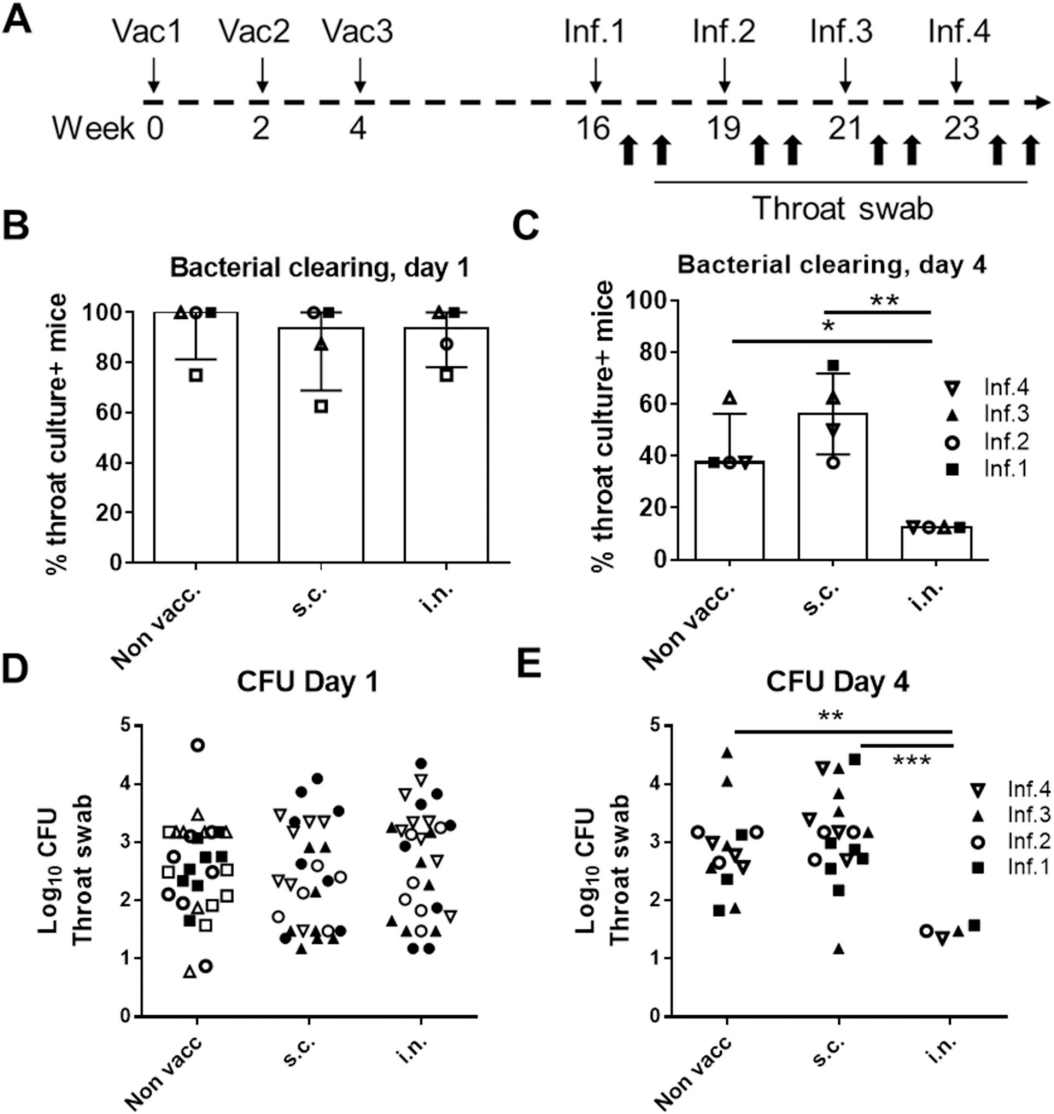
HGAS/CAF01 given s.c. does not induce protection against an intranasal infection. **A**. Vaccination and infection sch edule (‘Vac’:Vaccine, ‘Inf.’:Infection, ‘CFU’:Colony forming unit). Groups of female CB6F1 mice (n = 8) were vaccinated with HGAS formulated in CAF01 either via the s.c. or i.n. route. Mice were then rested before receiving repeated i.n. infections of 10^6^ MGAS5005 bacteria at week 16, 19, 21 and 23. After each infection bacterial numbers were determined in throat swabs. Bacterial numbers were very similar for the four infections and are therefore combined into the same plots (for individual experiments see S3 Fig). The percentage of throat culture positive animals are shown at day 1 **(B)** and at day 4 **(C)** post infection. The bacterial numbers in throat culture positive animals are shown in **(D)** at day 1 and **(E)** at day 4 post infection. Statistical significance was evaluated by an ANOVA followed by Tukey’s multiple comparison test using GraphPad Prism version 6.05. *p<0.05, **p<0.01, ***p<0.001 and ****p<0.0001.

Having demonstrated protection with a parenteral HGAS/CAF01 vaccine against a systemic GAS infection we next examined if this systemic immunity would also protect against an i.n. infection. We used a repeated low dose infection model to 1) address whether protection could be maintained over the course of multiple sublethal infections, 2) to use a model that reflect the human situation where repeated infections are common and 3) to use a model better suited for allowing T cells to play a protective role. Although an intranasal infection with GAS led to bacterial numbers in both throat swap, NALT (and nasal fluid) (S3 Fig), we only monitored the bacterial numbers in throat swaps in the vaccine experiments. Mice were vaccinated three times with two weeks interval with the HGAS/CAF01 vaccine, either via the s.c. or i.n. route, and infected with a non-lethal (10^6^ CFU) infection dose via the i.n. route at week 16, 19, 21 and 23 (Fig 2A). Following each of the infections, the bacteria in throat swaps were cultured and the numbers determined at day 1 and 4. As this model use a non-lethal low dose infection some animals in all groups were able to clear the infection by day 4.

Fig 2B–2E shows all the day 1 and 4 CFU data, each combined into one plot (individual CFU data after each of the infections were very similar as shown in S3 Fig) At day 1 after infection, we observed the same percentage of CFU positive animals (~90%) in all the groups (Fig 2B) indicating successful initial inoculation. Furthermore, the bacterial levels in the CFU positive animals were also similar at day 1 post infection (Fig 2D). In contrast, at day 4 the group of i.n. vaccinated mice vaccinated showed significantly reduced percentage of CFU positive animals (Fig 2C). The bacterial levels in CFU positive animals (of which there was fewer in the i.n. group due to increased protection/bacterial clearance in this group) was also significantly reduced in the i.n. immunized mice (Fig 2E, p<0.001). No CFU reduction was observed in mice vaccinated via the s.c. route after any of the four infections (Fig 2E and S3 Fig). In addition, despite having received several non-lethal infections, the non-vaccinated animals did apparently not develop a protective immune response in this model. Thus, only mice vaccinated via the i.n. route showed protection against i.n. infection with GAS.

### Comparing the initial recall response in s.c. and i.n. immunized mice following intranasal infection

To further explore why animals immunized by the parenteral route were not protected against an i.n. infection with GAS (in contrast to i.n. vaccinated mice), we next examined the mucosal immunity induced by the two administration routes before and after infection, at an early timepoint where infection induced natural immunity was still minimal.

**Fig 3:**
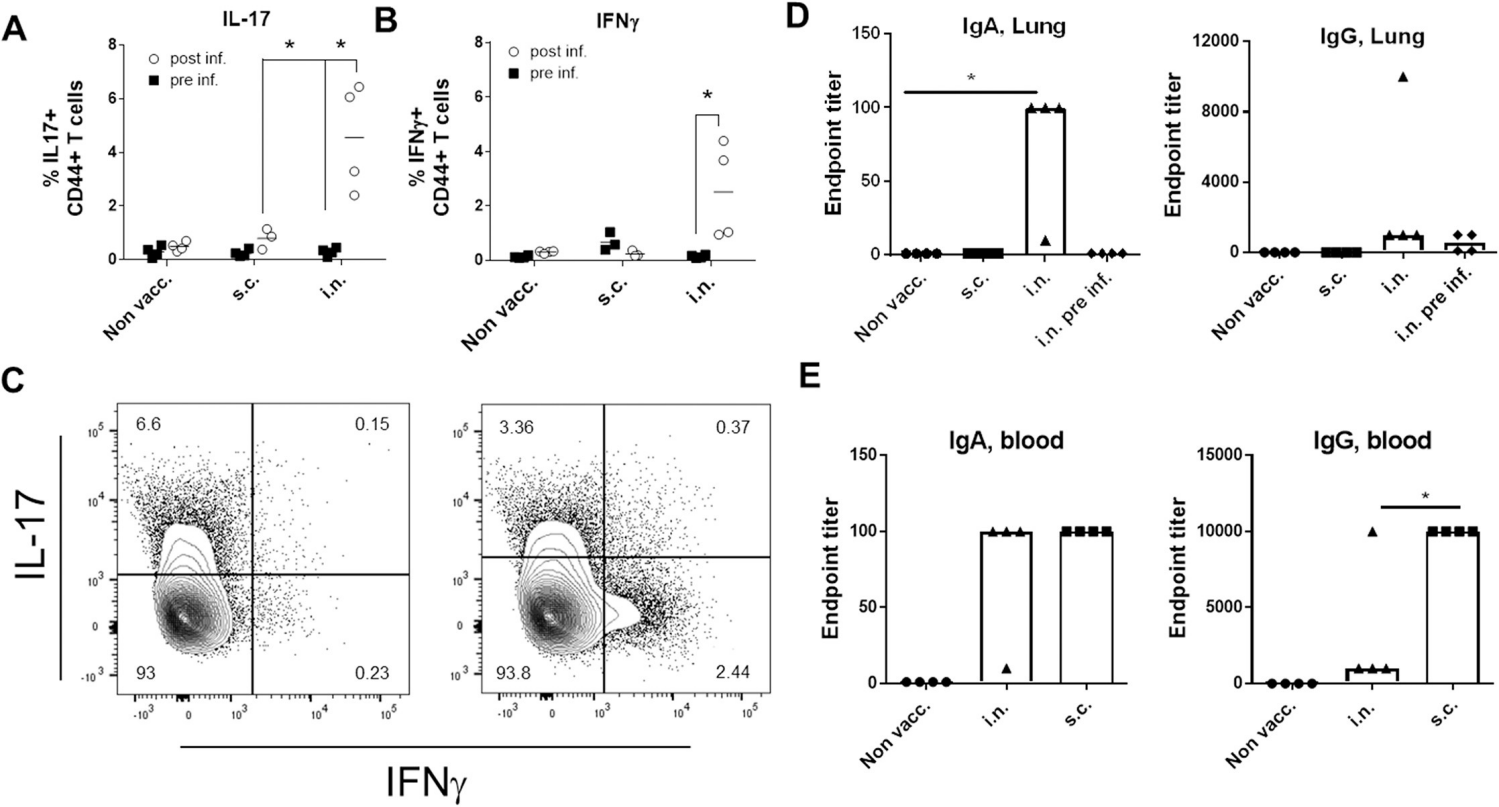
S.c. immunized animals do not develop recall responses in the lungs. Groups of female CB6F1 mice (n = 7/8) were vaccinated with HGAS/CAF01 either via the s.c. or i.n. route. Mice were rested for 26 weeks before an i.n. infection with 5 x 10^7^ MGAS5005 bacteria. Four mice were sacrificed right before infection while the remaining mice were sacrificed 7 days post infection. Lungs were harvested and cytokine expression in CD4 T cells was evaluated by ICS for IL-17 **(A)** and IFNγ **(B)**. A and B show two pooled experiments. All of the cytokine producing CD4 T cells were of a CD44 high phenotype. For complete gating strategy see S4 Fig **C.** Two examples of CD4^+^CD44^+^ cells from lung of the the i.n. vaccinated mice. The plots show the IL-17/IFNγ expression. **C** and **D**. Antibody responses measured by antigen-specific ELISA from the same experiment in lungs and blood. Lungs were perfused, homogenized and IgG and IgA responses were analyzed in the supernatants the day before infection (pre-inf, only shown for lungs)) and 7 days post infection. Statistical significance was evaluated by an ANOVA followed by Tukey’s multiple comparison test using GraphPad Prism version 6.05. *p<0.05, **p<0.01, ***p<0.001 and ****p<0.0001.

Mice were given the vaccine at week 0, 2, and 4, via the s.c. or i.n. route before receiving a high dose (5 x 10^7^ MGAS5005) i.n. challenge. Following infection, there was a significant increase in IL-17^+^ CD4 T cells and a smaller decrease in IFNγ^+^ cells in mice vaccinated via the i.n. route (Fig 3A and 3B. Gating strategy is shown in S4 Fig). The majority of cells were single expressors of either IL-17 or IFNγ without cytokine co-expression (Fig 3C). In addition, we observed that IgA titers dramatically increased in the lungs of i.n. vaccinated animals after infection recall (Fig 3D). In contrast, we did not measure any IgA in the lungs of s.c. vaccinated animals before or after infection. Mice that were s.c. vaccinated (but not i.n. vaccinated) showed a high IgG serum titer that was not increased following the intranasal infection (Fig 3E and S5 Fig). Compared to i.n. vaccinated mice, s.c. vaccinated mice also showed a higher Th17/Th1 response in the blood (S5 Fig), which was not reflected in the (perfused) lungs.

Taken together, infection with GAS led to a significant recall response in i.n. vaccinated mice, which was characterized by increased levels of IL-17 producing T cells (and to a slightly lesser degree, IFNγ) as well as increased mucosal levels of IgA. In contrast to a s.c. vaccination, an i.n. vaccination also induced a small increase in mucosal IgG that however only showed a minor increase following the infection (Fig 3D). The Th17/IgA recall response was observed despite a contraction of the post vaccine immune response to baseline levels due to the long resting period after the vaccination.

**Fig 4:**
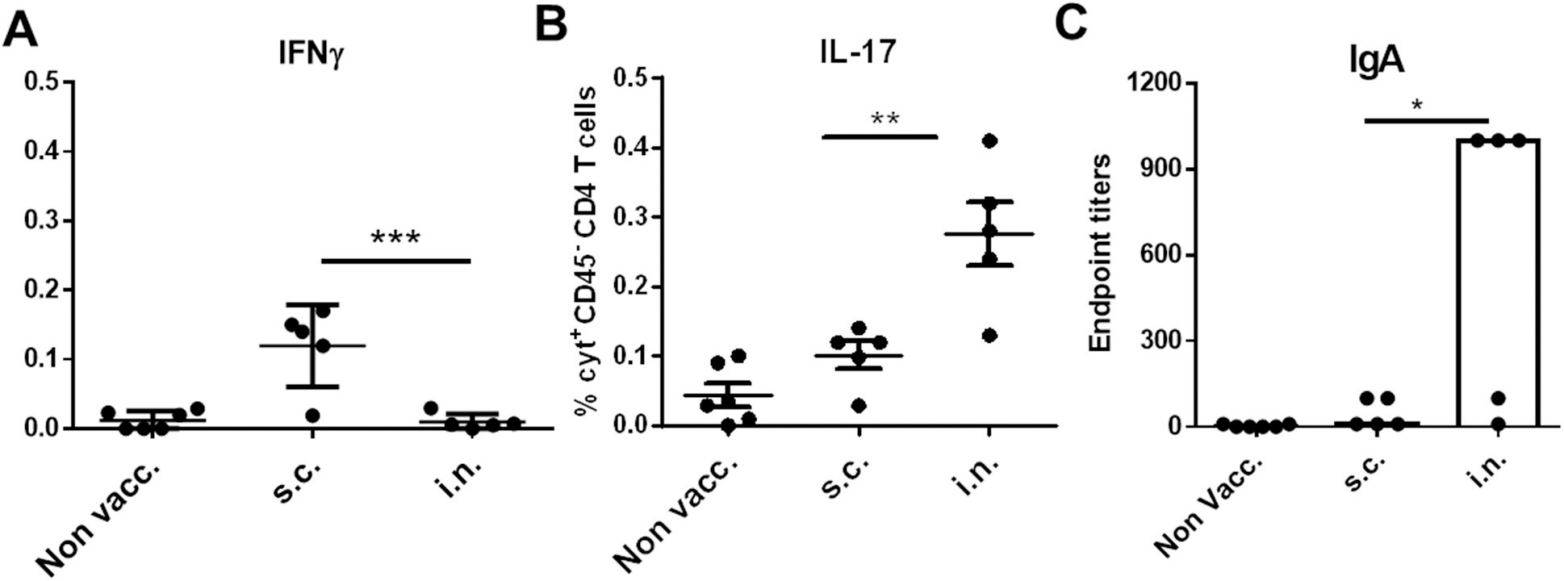
Lung responses, 72 hours post infection. Groups of six female CB6F1 mice were vaccinated with HGAS formulated in CAF01 either via the s.c. or i.n. route. Lungs were harvested at day 3 post infection and cytokine expression in CD45.2^-^, CD4^+^ T cells was evaluated by ICS for IL-17 **(A)** and IFNγ **(B)**. 250μl of the aCD45.2 solution was injected i.v. into each mouse via the tail vein, three minutes before euthanization of the mice. All of the cytokine producing CD4 T cells were of a CD44 high phenotype. **C.** Lungs were perfused, homogenized and IgA responses were analyzed in the supernatants 3 days post infection. Statistical significance was evaluated by an ANOVA followed by Tukey’s multiple comparison test using GraphPad Prism version 6.05. *p<0.05, **p<0.01, ***p<0.001 and ****p<0.0001.

We next expanded our findings of an early recall response in i.n. vaccinated mice by looking as early as day 3 post infection. Furthermore, we decided to also include an analysis to effectively distinguish between T cells localized in the lung parenchyma and T cells present in the lung vasculature by subjecting the mice to *in vivo* intravascular staining. This was conducted by injecting FITC-labelled anti-CD45 mAb intravenously (iv.CD45) three minutes before euthanization of the mice, thereby staining all intravascular, but not parenchymal lymphocytes with FITC, as described by Anderson et al. [25]. Thereafter lung cells were analyzed by flowcytometry. Fig 4A depicts the percentage of IL-17 and IFNγ producing T cells in the iv.CD45-ve CD4 populations. The data showed that only i.n. vaccinated mice experienced an increase in the percentage of Th17 T cells early after infection. In contrast, s.c. vaccinated mice showed a small increase in IFNγ producing T cells (Fig 4B). This was a transient increase since it was not observed at day 7 post infection (Fig 3). IgA was also increased, but only in i.n. vaccinated mice (Fig 4C).

Thus, i.n. vaccinated mice experienced an early recruitment of Th17 (Th1) T cells into the lung parenchyma post infection and an increase in lung IgA titer. This selective increase in Th17/IgA was observed at both day 3 and 7 post an intranasal infection with GAS.

### A vaccine strategy to induce both mucosal and systemic immunity

The presented data showed that parenteral immunization effectively protects against systemic infection whereas intranasal infections require intranasal vaccination. The data also demonstrated that a parenteral and an intranasal administered vaccine are selective concerning the location of the induced immunity, and that neither can induce both a strong mucosal and systemic immunity. Moreover, our data also indicated that protective immunity at both the systemic and mucosal location is required to protect against both mucosal and invasive infections. In an effort to induce both systemic and mucosal immunity with one vaccine strategy, we examined if co-administration of nasal and parenteral immunization in the same immunizations schedule would lead to strong immunity at both locations.

**Fig 5:**
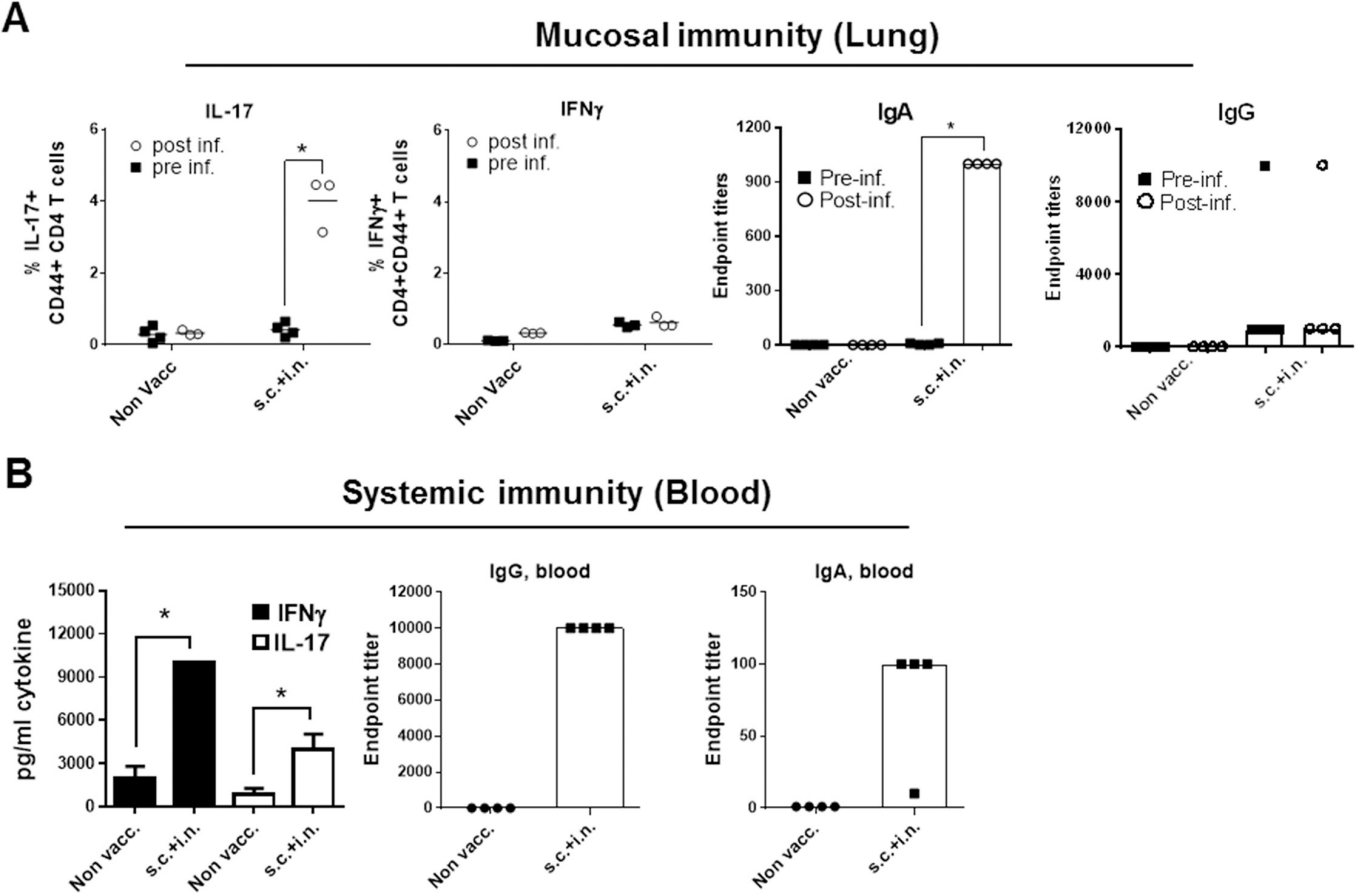
A combined intranasal and subcutaneous vaccination strategy to achieve both mucosal and systemic immunity. Groups of female CB6F1 mice (n = 6) were vaccinated with HGAS/CAF01 formulated in CAF01 either via s.c.+i.n. route. Mice were then subjected to an i.n. infection with 5 x 10^7^ MGAS5005 bacteria. At day 7 post infection lungs were harvested and IL-17 and IFNγ cytokine expression in CD44^+^CD4^+^ T cells was evaluated by ICS and IgA antibody responses in the lungs was measured by antigen-specific ELISA **(A).** IgG levels (endpoint titers were determined in serum and PBMCs were stimulated with HGAS for 72 h before measuring levels of IFNγ and IL-17 in culture supernatants by ELISA **(B).** The sc+in group was part of the experiment shown in Fig 3 and the non-vaccinated control group are therefore shared. Statistical significance was evaluated by an ANOVA followed by Tukey’s multiple comparison test using GraphPad Prism version 6.05. *p<0.05.

Mice vaccinated an simultaneously with an s.c. and an i.n. vaccine “s.c.+i.n.” at week 0, 2, and 4 received a high dose (5 x 10^7^ MGAS5005) i.n. challenge. At day 7 post infection lungs were carefully perfused to remove the blood, and analysed for recruitment of Th17 T cells and the presence of IgA. The data clearly showed that adding intranasal immunization to the parenteral immunization led to a strong post-infection increase in both the Th17 (CD4^+^CD44^+^IL-17^+^) and Th1 (CD4^+^CD44^+^IFNγ^+^) response as well the IgA response compared to the pre-infection response (Fig 5A). Mucosal IL-17 producing T cells were primarily IL-17 single expressers and did not co-express IFNγ/IL-2/TNFα (data not shown). Moreover, the s.c.+i.n. group also showed a strong systemic cellular immune response, measured by IFNγ or IL-17 secretion by *in vitro* antigen stimulated blood T cells (Fig 5B). Thus, by adding an intranasal immunization to a parenteral vaccination strategy it was possible to obtain both mucosal and systemic immunity.

## Discussion

The primary goal for this study was to examine whether systemic immunity could cross-protect against an intranasal infection. The results clearly showed that despite inducing protection against a intraperitonal infection, the systemic protective anti-GAS response induced by the parenteral vaccine did not offer protection against an i.n. infection. We used a repeated infection model where the mice receive several sub lethal infections. In our model, the mice were subjected to four consecutive infections, and at day 1 after each of the infections ~90% of mice in all groups were colonized with bacteria in the throat, which confirmed successful infection. Four days later there was a decrease in 1) the number of mice with a positive throat culture and 2) the CFU levels in mice that had received vaccination by the i.n. route. In contrast, mice vaccinated by the s.c. route were (despite harboring protective immunity against a systemic infection) unable to demonstrate improved control over the 4 sublethal intranasal infections compared to non-vaccinated mice (Fig 2). These data imply that parenteral immunization is not suitable for inducing protective mucosal immunity. Providing a possible explanation for this, follow-up experiments demonstrated that parenterally vaccinated mice failed to mount a Th17/IgA recall response in the lungs, which was in contrast to the i.n. group (Figs 3 and 4). This is in agreement with studies showing that lymphocytes primed in the lungs have strong tropism to the airways, whereas parenteral immunization primarily induce T cells that lack the mucosal homing markers [26–28]. Interestingly, in an early human clinical trial using M protein, patients who were immunized intranasally with the M protein had both reduced throat colonization and clinical illness compared with patients vaccinated systemically [24]. Taken together, with our own data, this indicates the importance of a local immune response in GAS protection and suggest that the intranasal/mucosal administration may be the most optimal in preventing an intranasal infection with GAS. The local immunity consisted primarily of Th17/IgA immunity. However, IgG was also observed in i.n. vaccinated mice and a protective role for IgG can not be excluded.

Although the first experiment in our study only aimed at confirming the protective efficacy of a parenteral model vaccine against a systemic infection, it actually indicated a role for Th1/Th17 immunity and that strong humoral immunity may not be sufficient for protection against a systemic infection. Thus, mice vaccinated s.c. with HGAS formulated in the Th1/Th17 inducing adjuvant CAF01 [1, 29] showed elevated IFNγ and IL-17 responses compared to mice vaccinated with non-adjuvanted HGAS. However, antibody responses measured in serum were almost identical in the two groups of mice. After receiving a lethal homologous GAS challenge, all the non-vaccinated mice had to be euthanized within the first 14 hours. Shortly after, mice vaccinated with non-adjuvanted HGAS started to show severe clinical signs of disease and only one mouse survived after 24 hours. This correlated with high bacterial numbers in the spleen. Thus, despite a high IgG response in the HGAS vaccinated group, these mice failed to control the systemic infection, suggesting that antibodies alone may not be sufficient to control the infection, although it cannot be fully excluded that the fine specificity of the IgG response was different in the HGAS vaccinated animals. Using another adjuvant, CpG, also showed a selective increase in Th1/Th17 immunity, and 100% survival (S2 Fig). This is in contrast to studies showing that passive transfer of immune serum can protect against a lethal infection [30–32]. However, in these studies, large volumes of serum were injected into the peritoneum 24 hours before i.p. infection and the bacteria were therefore immediately introduced to an environment with high levels of serum antibodies. Such studies might therefore represent a scenario that can never be reached with active immunization. It has also been shown that parenteral vaccination increases serum IgG levels, which is believed to confer protection against lethal GAS infection [7, 33–37], but these studies did not characterize the cellular immune responses and it can therefore not be excluded that cellular responses contributed to immunity. Several studies have indicated a protective role for Th17 cells against an i.n. GAS infection [21–23], and that Th17 are induced by an i.n. GAS infection [22, 38], and together with our data, this suggests that protection against GAS can be improved compared to what is offered solely by antibody responses by using a strong Th1/Th17 inducing adjuvant. IL-17 might directly enhance bacterial clearance by promoting fast recruitment of neutrophils to site of infection as has been demonstrated by injecting recombinant IL-17 into the peritoneal cavity [39]. IL-17 may also be (indirectly) important for induction of IgA at mucosal sites [40, 41], and we did observe a correlation between lung Th17 cells and IgA. It will be important to determine in future experiments if the IgA was produced by local tissue resident B cells.

Taken together, although systemically primed Th1/Th17 T cells did not protect against an i.n. infection, our data nevertheless suggest that Th1/Th17 T cells are important in the defense against both a systemic and an intranasal infection, but that they have to be primed in the infected compartment/location. Alternatively, they have to be ‘pulled’ to the infected compartment shortly after their priming. Our data indicate that this can be accomplished by adding an i.n. immunization to a vaccination strategy that already includes s.c. vaccinations. This is in agreement with the ‘prime-pull’ vaccine strategy that we recently published where systemically primed Th17 T cells could be ‘pulled’ to a mucosal site with a mucosal booster vaccine and increase the effect of the mucosal vaccine [42]. That study was conducted with the recombinant GAS protein ScpA, and due to the low immunogenicity of recombinant proteins, and the fact that the intranasal site is more difficult to prime an immune response in, we find that the s.c./i.n. strategy is particular well suited to induce a vaccine specific mucosal response against low immunogenic recombinant proteins [42]. Finally, the vaccine generated recalled Th17 cells did not co-express IFNγ, IL-2 or TNFα. As Th17 have been implicated in pathological reactions and autoimmunity it will be important to establish if this only applies for some subtypes of Th17 cells, and in particular those generated by the infection and not the vaccine. In fact, it has been suggested that Th1 and Th17 have been implicated in the autoimmune process leading to the formation of heart lesions in rheumatic heart disease (RHD) (6) and that Th17 T cells expressing IFNγ may be pathogenic [21].

In our repeated infection model we noticed that animals did not develop resistance against a third or fourth infection despite having been subjected to several preceding infections. Thus, it can be speculated that the immunity induced by GAS exposure (i.e. infection driven immunity) is suboptimal against a subsequent infection, among other things due to poor local cellular anti-GAS immunity. This observed lack of infection induced protective immunity is in contrast to a previous study that indicated that infected mice develop protective immunity [22]. However, this discrepancy might be explained by the shorter resting time in the study by Wang and co-workers between the last sublethal infection and the high-dose lethal infection (2 days compared to 1–3 month in our study) [22].

In conclusion, our results showed that that local priming of the immune response is crucial for protection, and furthermore indicated a protective role for mucosal Th17/IgA immunity. As protection against an intranasal infection with GAS is a requirement for any GAS vaccine, a parenteral vaccine strategy may not be sufficient to provide protection. Furthermore, the importance of Th1/Th17 immunity also exclude a Th2 inducing adjuvant, such as Aluminum hydroxide, as the obvious choice for an adjuvant. This is further supported by a our recent publication showing that anti-GAS immunity in humans is dominated by Th1/Th17/IgG3 immunity [15].

## Supporting information

S1 Fig Clinical scores of individual infected animals. Six weeks after the last immunization, mice (n = 5) were challenged with a lethal dose of MGAS5005 bacteria (1–1.5 10^7^ CFU/mouse) by i.p. injection. Mice were monitored over a period of 24 hours according to a validated clinical scoring system from 0–4. The scores for individual animals are shown. Mean values are shown in Fig 1.
(TIF)

S2 Fig CpG induce immune responses similar to CAF01 and confer protection. **A and B**. Eight Female CB6F1 mice were vaccinated 3 x s.c. with two weeks interval with heat inactivated GAS either alone or in CpG. Two weeks after the last immunization three animals were sacrificed and PBMcs were stimulated with HGAS for 72h before measuring levels of IFNγ and IL-17 in culture supernatants by ELISA. **C**. Serum IgG was analysed by antigen-specific ELISA **D and E.** Six weeks after the last immunization, the remaining mice (n = 5) were challenged with a lethal dose of MGAS5005 bacteria (1–1.5 x 10^7^ CFU/mouse) injected into the peritoneum. Mice were monitored over a period of 24 hours according to a validated clinical scoring system from 0–4. When reaching a score of 4, mice were euthanized and data was plotted in Kaplan-Meier survival curves. **p = .0023 between the non-vaccinated and CpG group with a Chi-square test. All the animals survived in the CpG group, which correlated with a lower colonization of the spleen (E).
(TIF)

S3 Fig Bacterial numbers in throat swabs after repeated i.n. infections. **A-F**. Groups of female CB6F1 mice (n = 8) were vaccinated with HGAS formulated in DDA/TDB either via the s.c. or i.n. route. Mice then received repeated i.n. infections of 10^6^ MGAS5005 bacteria (M5005) at week 16 (A), 19 (B), 21 (C) and 23 (D). After each infection bacterial numbers were determined in throat swabs. Individual mice are shown. **E and F.** Following intranasal infection of CB6F1 mice (n = 3–5) with MGASM5005 GAS strain or a M5 Manfredo strain, CFU was determined in NALT, Lung and wash nasal fluid at day one (2 h) and day 2 (24 h) post the first infection. **G**. CFU in MGASM5005 infected mice was compared in throat swab and nasal wash fluid. **H**. IgA levels were determined in throat swab at day 1 post intranal infection with 10^6^ MGAS5005.
(TIF)

S4 Fig Gating tree for identification of IL-17 and IFNγ producing CD4 T cells. Lung cells of mice immunized i.n. with H-GAS were analyzed 7 days post infection with GAS. Singlets were identified by their forward scatter (FSC) peak height (H) and area (A). Lymphocytes were gated based on their FSC vs. side scatter (SSC) profile and the CD4 T cell population was further devided into CD44hi subsets producing IL-17 and IFNγ. Data is shown from cells cultured in medium alone (None) or stimulated with H-GAS.
(TIF)

S5 Fig Systemic immune responses following an s.c. or i.n. vaccination. **A and B**. Groups of female CB6F1 mice (n = 12) were vaccinated twice with HGAS formulated in CAF01 (at 3 weeks interval) either via the subcutaneous (s.c.) or intranasal (i.n.) route as indicated. 4 weeks after the final vaccination cytokine expression was evaluated by ELISA after in vitro stimulation with HGAS for IFNγ and IL-17 for 72 hours. Graph shows mean +/- SEM. ANOVA followed by Tukey’s multiple comparison test using GraphPad Prism version 6.05. *p<0.05, **p<0.01, ***p<0.001 and ****p<0.0001. **C.** IgG isotypes were measured in pooled serum after infection.
(TIF)

S6 Fig Recall responses in the lungs of GAS infected mice. Groups of five female CB6F1 mice were vaccinated with HGAS/CAF01 by the route indicated. Mice were then subjected to an i.n. infection with 5 x 107 MGAS5005 bacteria. Two mice were sacrificed right before infection while the remaining 3 mice were sacrificed 7 days post infection. Lungs were harvested and cytokine expression in CD4 T cells was evaluated by ICS for IL-17 (A) and IFNγ. All of the cytokine producing CD4 T cells were of a CD44 high phenotype. For complete gating strategy see S4 Fig. The plots show the IL-17/IFNγ expression. Statistical significance was evaluated by a t-test using GraphPad Prism version 6.05 where p<0.05 was considered significant.
(TIF)
