# Supplementary figures and images for: Local Th17/IgA immunity correlate with protection against intranasal infection with Streptococcus pyogenes
Source: PLoS One. 2017 Apr 17;12(4):e0175707. doi: 10.1371/journal.pone.0175707 (PMC5393599; doi:10.1371/journal.pone.0175707)

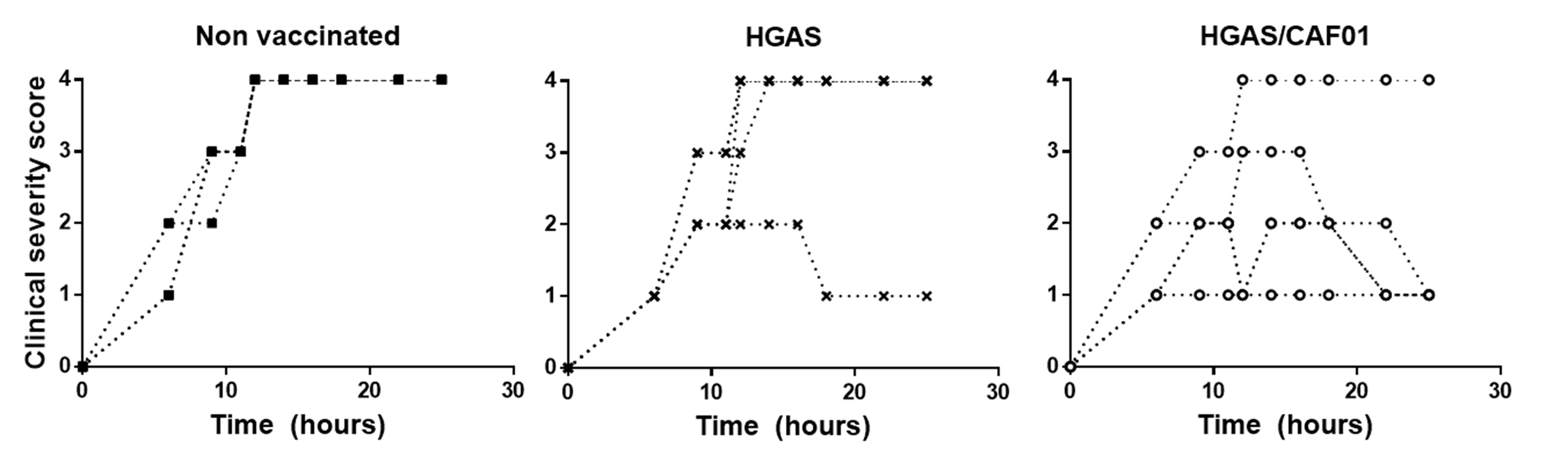

Supplement: S1 Fig — Six weeks after the last immunization, mice (n = 5) were challenged with a lethal dose of MGAS5005 bacteria (1–1.5 107 CFU/mouse) by i.p. injection. Mice were monitored over a period of 24 hours according to a validated clinical scoring system from 0–4. The scores for individual animals are shown. Mean values are shown in Fig 1. (TIF) [file pone.0175707.s001.tif]

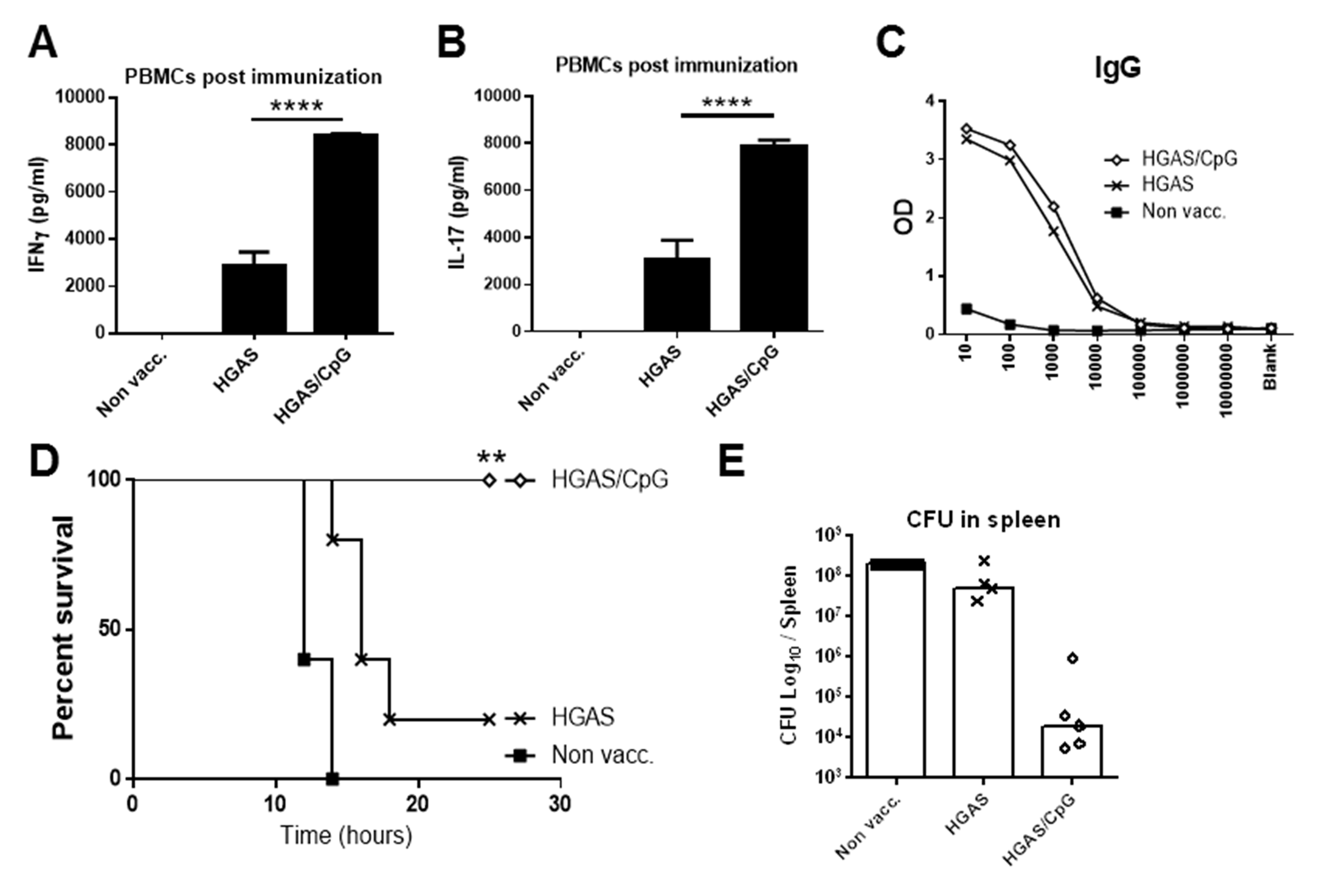

Supplement: S2 Fig — A and B. Eight Female CB6F1 mice were vaccinated 3 x s.c. with two weeks interval with heat inactivated GAS either alone or in CpG. Two weeks after the last immunization three animals were sacrificed and PBMcs were stimulated with HGAS for 72h before measuring levels of IFNγ and IL-17 in culture supernatants by ELISA. C. Serum IgG was analysed by antigen-specific ELISA D and E. Six weeks after the last immunization, the remaining mice (n = 5) were challenged with a lethal dose of MGAS5005 bacteria (1–1.5 x 10^7^ CFU/mouse) injected into the peritoneum. Mice were monitored over a period of 24 hours according to a validated clinical scoring system from 0–4. When reaching a score of 4, mice were euthanized and data was plotted in Kaplan-Meier survival curves. **p = .0023 between the non-vaccinated and CpG group with a Chi-square test. All the animals survived in the CpG group, which correlated with a lower colonization of the spleen (E). (TIF) [file pone.0175707.s002.tif]

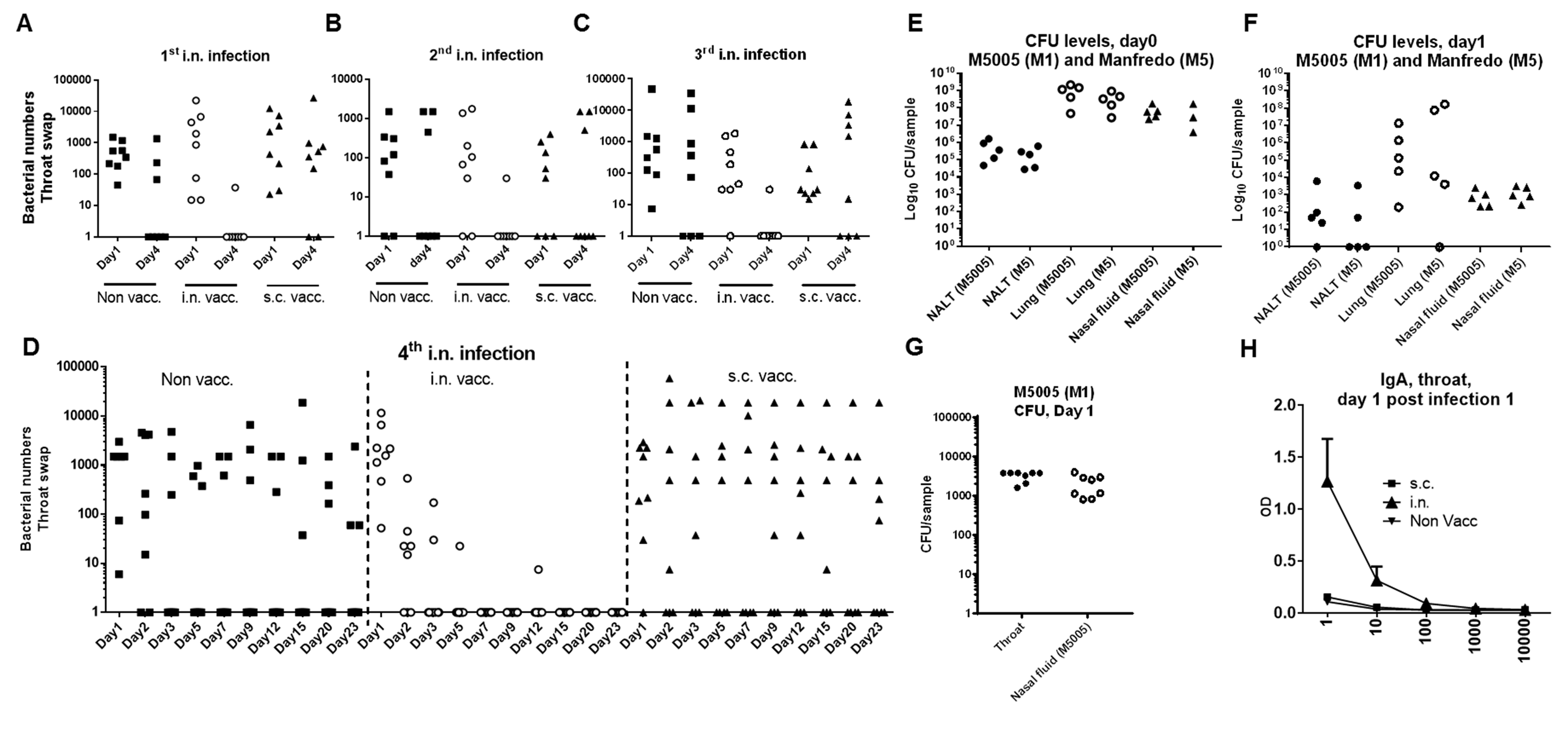

Supplement: S3 Fig — A-F. Groups of female CB6F1 mice (n = 8) were vaccinated with HGAS formulated in DDA/TDB either via the s.c. or i.n. route. Mice then received repeated i.n. infections of 106 MGAS5005 bacteria (M5005) at week 16 (A), 19 (B), 21 (C) and 23 (D). After each infection bacterial numbers were determined in throat swabs. Individual mice are shown. E and F. Following intranasal infection of CB6F1 mice (n = 3–5) with MGASM5005 GAS strain or a M5 Manfredo strain, CFU was determined in NALT, Lung and wash nasal fluid at day one (2 h) and day 2 (24 h) post the first infection. G. CFU in MGASM5005 infected mice was compared in throat swab and nasal wash fluid. H. IgA levels were determined in throat swab at day 1 post intranal infection with 106 MGAS5005. (TIF) [file pone.0175707.s003.tif]

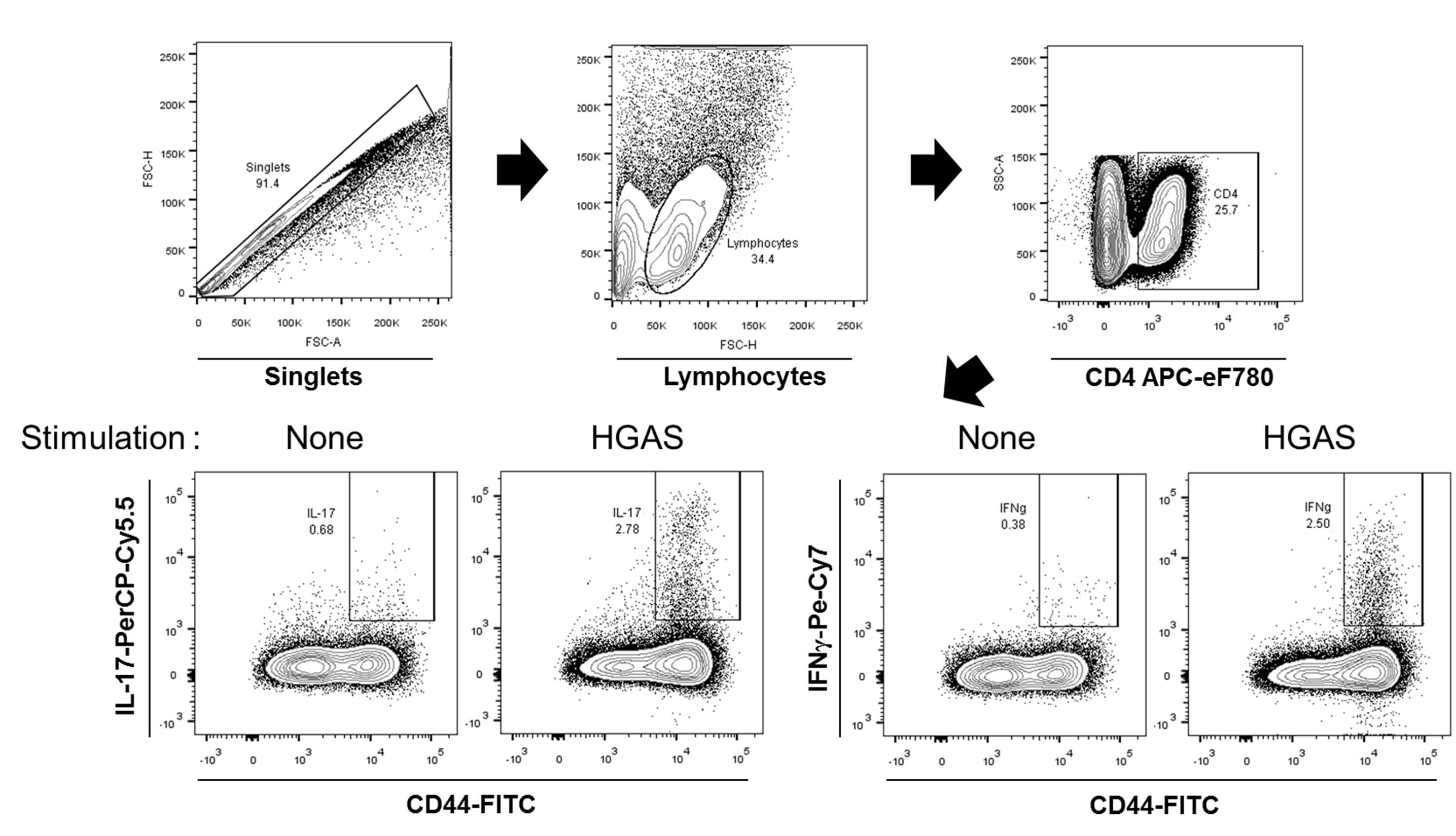

Supplement: S4 Fig — Lung cells of mice immunized i.n. with H-GAS were analyzed 7 days post infection with GAS. Singlets were identified by their forward scatter (FSC) peak height (H) and area (A). Lymphocytes were gated based on their FSC vs. side scatter (SSC) profile and the CD4 T cell population was further devided into CD44hi subsets producing IL-17 and IFNγ. Data is shown from cells cultured in medium alone (None) or stimulated with H-GAS. (TIF) [file pone.0175707.s004.tif]

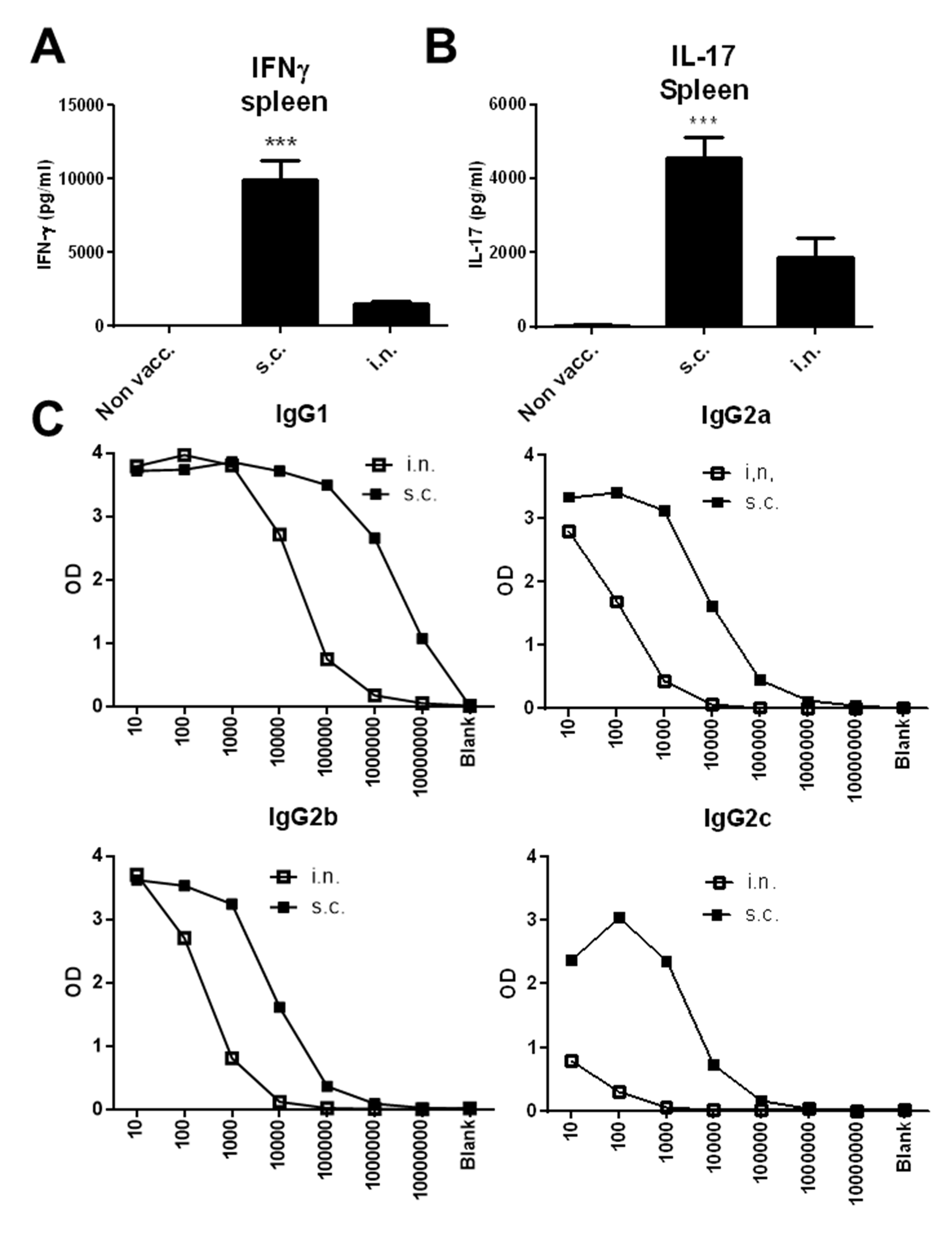

Supplement: S5 Fig — A and B. Groups of female CB6F1 mice (n = 12) were vaccinated twice with HGAS formulated in CAF01 (at 3 weeks interval) either via the subcutaneous (s.c.) or intranasal (i.n.) route as indicated. 4 weeks after the final vaccination cytokine expression was evaluated by ELISA after in vitro stimulation with HGAS for IFNγ and IL-17 for 72 hours. Graph shows mean +/- SEM. ANOVA followed by Tukey’s multiple comparison test using GraphPad Prism version 6.05. *p<0.05, **p<0.01, ***p<0.001 and ****p<0.0001. C. IgG isotypes were measured in pooled serum after infection. (TIF) [file pone.0175707.s005.tif]

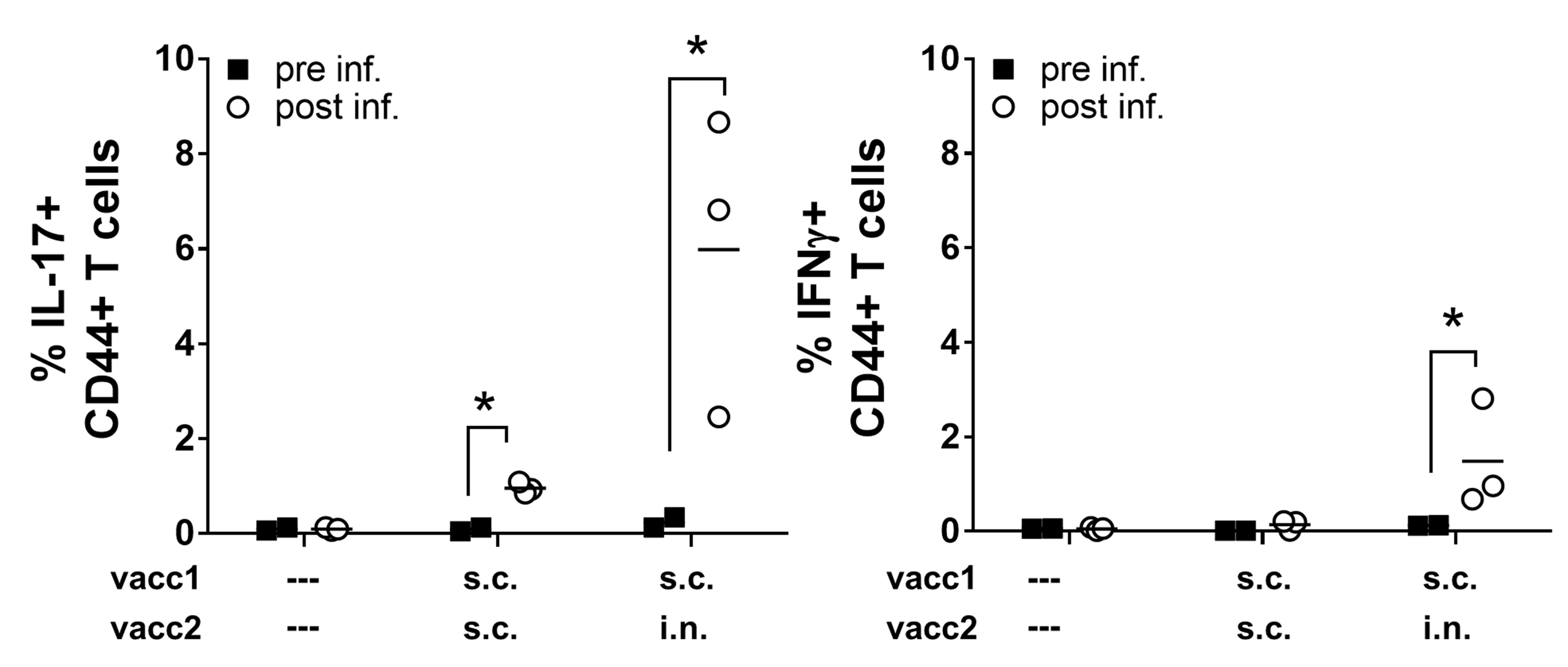

Supplement: S6 Fig — Groups of five female CB6F1 mice were vaccinated with HGAS/CAF01 by the route indicated. Mice were then subjected to an i.n. infection with 5 x 10^7^ MGAS5005 bacteria. Two mice were sacrificed right before infection while the remaining 3 mice were sacrificed 7 days post infection. Lungs were harvested and cytokine expression in CD4 T cells was evaluated by ICS for IL-17 (A) and IFNγ. All of the cytokine producing CD4 T cells were of a CD44 high phenotype. For complete gating strategy see S4 Fig. The plots show the IL-17/IFNγ expression. Statistical significance was evaluated by a t-test using GraphPad Prism version 6.05 where p<0.05 was considered significant. (TIF) [file pone.0175707.s006.tif]
